# Supplementary material for: In-host population dynamics of Mycobacterium tuberculosis complex during active disease
Source: eLife. 2021 Feb 1;10:e61805. doi: 10.7554/eLife.61805 (PMC7884073; doi:10.7554/eLife.61805)
Supplement: Transparent reporting form [file elife-61805-transrepform.pdf]

eLIFE
1st Floor
24 Hills Road
Cambridge CB2 1JP, UK
P 01223 855340
W elifesciences.org
T @elife

eLife's transparent reporting form

We encourage authors to provide detailed information *within their submission* to facilitate the interpretation and replication of experiments. Authors can upload supporting documentation to indicate the use of appropriate reporting guidelines for health-related research (see EQUATOR Network), life science research (see the BioSharing Information Resource), or the ARRIVE guidelines for reporting work involving animal research. Where applicable, authors should refer to any relevant reporting standards documents in this form.

If you have any questions, please consult our Journal Policies and/or contact us: editorial@elifesciences.org.

Sample-size estimation

- You should state whether an appropriate sample size was computed when the study was being designed
- You should state the statistical method of sample size computation and any required assumptions
- If no explicit power analysis was used, you should describe how you decided what sample (replicate) size (number) to use

Please outline where this information can be found within the submission (e.g., sections or figure legends), or explain why this information doesn't apply to your submission:

All whole-genome sequencing data analyzed in this study is publicly available (from ENA and NCBI) and was collected from previously published studies. Detailed information on the isolates sequenced including accession numbers & the source study for each isolate is available in Table S1-2. Sample size was chosen by including all high quality Mtb genome data from these studies.

Replicates

- You should report how often each experiment was performed
- You should include a definition of biological versus technical replication
- The data obtained should be provided and sufficient information should be provided to indicate the number of independent biological and/or technical replicates
- If you encountered any outliers, you should describe how these were handled
- Criteria for exclusion/inclusion of data should be clearly stated
- High-throughput sequence data should be uploaded before submission, with a private link for reviewers provided (these are available from both GEO and ArrayExpress)

Please outline where this information can be found within the submission (e.g., sections or figure legends), or explain why this information doesn't apply to your submission:

eLife Sciences Publications, Ltd is a limited liability non-profit non-stock corporation incorporated in the State of Delaware, USA, with company number 5030732, and is registered in the UK with company number FC030576 and branch number BR015634 at the address 1st Floor, 24 Hills Road, Cambridge CB2 1JP | August 2014

Data exclusions are described in the Results (sub-section: Identifying clonal Mtb populations in-host) and Methods (sub-section: Mixed lineage and contamination detection for longitudinal and replicate isolate pairs). The following has been taken from the Results, "To isolate ... dynamics ... among the 307 subjects ... we excluded 32 subjects with isolate microbiological contamination at any time point, and 31 subjects with evidence for mixed infection with two or more Mtb lineages (Fig. 1B, Fig. S1). We also excluded 44 subjects with evidence for re-infection with a different Mtb strain ... (Methods, Fig. 1C, Fig. S1)." Additionally, we used technical replicates to estimate the noise in comparing allele frequencies between pairs of sequenced isolates. This is described in the Results (sub-section: Genome-wide in-host diversity; Fig. 4).

Statistical reporting

- Statistical analysis methods should be described and justified
- Raw data should be presented in figures whenever informative to do so (typically when N per group is less than 10)
- For each experiment, you should identify the statistical tests used, exact values of N, definitions of center, methods of multiple test correction, and dispersion and precision measures (e.g., mean, median, SD, SEM, confidence intervals; and, for the major substantive results, a measure of effect size (e.g., Pearson's r, Cohen's d)
- Report exact p-values wherever possible alongside the summary statistics and 95% confidence intervals. These should be reported for all key questions and not only when the p-value is less than 0.05.

Please outline where this information can be found within the submission (e.g., sections or figure legends), or explain why this information doesn't apply to your submission:

Details on statistical analyses can be found in the Results Section (sub-sections: Allele frequency >19% predicts subsequent fixation of resistance variants; Determinants of antibiotic resistance acquisition and microbiological treatment failure; Characteristics of mutations in-host; Antibiotic resistance and PE/PPE genes vary while antigens remain conserved; PE/PPE variation is independent of T-cell recognition; Identifying candidate pathoadaptive loci from genome-wide variation) and the Methods Section (sub-sections: True & false positive rate analysis for heteroresistance mutations; Mutation density test; Data analysis and variant annotation).

(For large datasets, or papers with a very large number of statistical tests, you may upload a single table file with tests, Ns, etc., with reference to sections in the manuscript.)

Group allocation

- Indicate how samples were allocated into experimental groups (in the case of clinical studies, please specify allocation to treatment method); if randomization was used, please also state if restricted randomization was applied
- Indicate if masking was used during group allocation, data collection and/or data analysis

Please outline where this information can be found within the submission (e.g., sections or figure legends), or explain why this information doesn't apply to your submission:

There were no experimental groups in this study, data from different subjects was not allocated to different groups.

Additional data files ("source data")

- We encourage you to upload relevant additional data files, such as numerical data that are represented as a graph in a figure, or as a summary table
- Where provided, these should be in the most useful format, and they can be uploaded as "Source data" files linked to a main figure or table
- Include model definition files including the full list of parameters used
- Include code used for data analysis (e.g., R, MatLab)
- Avoid stating that data files are "available upon request"

Please indicate the figures or tables for which source data files have been provided:

Tables S1-S3 contain information on the source studies and public sequencing data used in this study. Table S6 contains the data used to generate Fig. 2 & Fig. S2. Table S7 contains the data used to generate Fig. 3. Table S8 & S13-S14 contains the data used to generate Fig. 5-6. Tables S9-S10 contain the data used to generate Fig. S5-S6. Table S11 contains the data used to generate Fig. S3. Table S12 contains the data used to generate Fig. S4. Tables S15-S17 contain the data used in the convergent evolution pathway analysis described in Results (sub-section: Identifying candidate pathoadaptive loci from genome-wide variation). Tables S18-S19 contain the data used to generate Fig. 7 & Fig. S10-S11. Table S20 contains the data used in the analysis for Supplementary Note 2.
